# Important statistical points to improve and promote the methodology of the articles on medical sciences, particularly nephrology and kidney; a review article

**DOI:** 10.12861/jrip.2015.02

**Published:** 2015-03-01

**Authors:** Ali Ahmadi, Hamid Soori

**Affiliations:** ^1^Department of Epidemiology and Biostatistics, School of Public Health, Shahrekord University of Medical Sciences, Shahrekord, Iran; ^2^Department of Epidemiology, School of Public Health, Shahid Beheshti University of Medical Sciences, Tehran, Iran

**Keywords:** Epidemiologic approaches, Epidemiologic research, Statistical analysis, Methodology, Statistical reporting

## Abstract

**Background:** Quality of articles’ methodology is one of the important factors which is considered by researchers.

**Objectives:** This study was conducted to determine statistical guidelines on promotion of methodology’s quality in the articles concerning medical sciences, particularly nephrology, to assist authors and reviewers.

**Materials and Methods:** This study is a systematic review. Initially, the keywords "Epidemiologic Methods/analysis" [Mesh] OR "Epidemiologic Methods/epidemiology" and "reporting" were selected in Medline database. Then, reliable databases were searched for relevant publications. Being relevant, containing viewpoints, and recommending statistical guidelines as well as approval of at least two of the three examiners of articles were determined as the inclusion criteria into the study.

**Results:** Two hundred relevant articles were retrieved. Thirty-two articles met the inclusion criteria. By the examined articles, 30 applied points have determinative role for improving and promoting quality of articles methodology. Of the important points, introducing and describing target community and statistical population, mentioning article title, introducing independent and dependent variables as well as confounders, reporting sample size for subgroups and the whole study, summarizing the data according to their statistical distribution (reporting mean and standard deviation for data with normal distribution), reporting the type of rate (incidence, survival), ratio (odds, hazard) or risk (absolute, relative, difference) with 95% CI and the used software could be mentioned.

**Conclusion:** The most important factors contributing greatly to the quality of articles’ methodology on nephrology were reported in the present study. Applying these factors by articles authors and reviewers could lead to improve articles’ and journals’ quality. In addition, use of the findings of the present study in articles’ materials and methods could avoid research errors.

***Implication for health policy/practice/research/medical education*:**

Prevalent errors in articles on medical sciences and offering guidelines to avoid these errors are regarded as important responsibilities of experts on methodology and materials and methods. Use of these factors by authors, particularly reviewers, could lead to improving articles’ and journals’ quality.

## Introduction

Scientific and peer-reviewed journals are considered as one of the most important instruments to inform and expand the knowledge of scientists and researchers. These journals are regarded an important index for knowledge generation and transmission. The quality of these journals and the articles appearing in them is one of the important factors which is paid attention by scientometric institutes to rank knowledge generation in the world (1). The quality of statistical reports in medical texts has attracted researchers’ attention since 1966. Since 1988, important works have appeared in this regard (2,3). Since then, different and several studies on this subject have been published. The well-known paper by Professor Altman has also offered helpful guidelines for authors and researchers in medical sciences (4). In examining and assessing the published articles in these journals, numerous errors in results, materials and methods, and methodology are still being seen. Statistical errors in medical journals of China were reported more than 80% and in International British Journal of Psychology, statistical errors were reported up to 40% (5). In Iran, these errors are common, as well. Of common errors in articles authored by Iranian researchers, errors in reporting results, composing materials and methods and analyzing data, enrolling samples, calculating sample size, randomizing, blinding, obtaining informed consent when necessary, calculating and reporting confidence interval for effect and association rates, and drawing graphs and tables could be mentioned (6,7). Therefore, particular attention to common and prevalent errors in articles on medical sciences and offering guidelines to avoid these errors are regarded as important responsibilities of experts on methodology and materials and methods.

## Objectives

This study was conducted to determine statistical guidelines on promotion of methodology’s quality in the articles concerning medical sciences, particularly nephrology, to assist authors and reviewers.

## Materials and Methods

This study is a systematic review. Initially, the keywords “*Epidemiologic Methods/analysis” [Mesh] OR “Epidemiologic Methods/epidemiology*” and “*reporting”* were selected in Pubmed database. Using a previous assessment of the methodology in Iranian scientific, research journals (8), we searched for the relevant publications in authentic databases (PubMed, Scopus, Ovid, Google Scholar) through terms of statistical reports, research methodology, how to conduct a research in medical sciences and to report results, statistical analysis, materials and methods, method of analysis, epidemiologic studies, nephrology, and statistical analysis. More than 200 articles were retrieved. After examination, 32 articles had the inclusion criteria and were relevant (2-33). The articles were studied and their useful guidelines and key points were written down to avoid statistical errors (26,33). Designing and implementation of the study was shown in Box 1. To finalize important statistical points to improve the quality of published articles on medical sciences, particularly, kidney and nephrology, valid checklists of consolidated standards of reporting trials (CONSORT), reporting recommendations for tumor marker prognostic studies (REMARK), standards for reporting of diagnostic accuracy (STARD), consolidated criteria for reporting qualitative research (COREQ), strengthening the reporting of observational studies in epidemiology (STROBE), preferred reporting items for systematic reviews and meta-analyses (PRISMA), enhancing transparency in reporting the synthesis of qualitative research (ENTREQ), consolidated health economic evaluation reporting standards (CHEERS), statistical analysis and methods in the published literature (SAMPLE), and consensus-based clinical case reporting (CARE) were employed. These checklists are prepared and made available by Cochrane institute entitled methodological expectations of Cochrane intervention reviews (MECIR) and enhancing the quality and transparency of health research (EQUATOR) network.

## Results

By the examined texts, 30 applied points contributed greatly to promoting articles methodology. Failure to mention the type of conducted study in methodology section and failure to report confidence interval for rates and ratios, as well as to report the employed analyses briefly and unclearly and failure to specify sample size in study subgroups were common defects of articles related to medical sciences. Paying attention to recommendations of *International Committee of Medical Journal Editors*, including detailed and adequate description of statistical methods used in materials and methods, so that, if the reader access the crude data and analyze them, he/she will obtain similar, consistent results; introducing the

**Box 1: Stepwise of the study (designing and implementation)**

1. Searching in PubMed and selecting appropriate keywords existing in Mesh
2. Establishing strategy for searching (combining appropriate keywords and accessible databases)
3. Searching in databases including PubMed, Scopus, ScienceDirect, Ovid, and Google Scholar
4. Enlisting the retrieved texts by the time of publication, abstracts and full texts of the articles (n=200)
5. Examining the articles for inclusion criteria and methodology (160 articles excluded and 32 articles judged as meeting inclusion criteria)
6. Writing down the recommendations and key points offered by the articles for promoting quality of articles methodology
7. Complying extracted guidelines with referenced checklists and reliable resources, and finalizing list of guidelines and statistical points

statistical methods used for crude data and preparing the data for analysis; for example, how non-normal distribution of the data on dependent variables has been turned into normal, classifying and merging quantitative data like age, blood pressure, and gender; describing the purpose of analysis clearly and doing required analyses and describing target community and sample population; identifying and introducing independent, dependent (response), and confounding variables and describing and summarizing them with common, suitable statistics like mean and standard deviation; reporting how sample size has been calculated for subgroups and in whole; reporting and defining numerator and denominator of calculated proportion and describing percentage; summarizing and reporting the data which have approximately normal distribution with mean and standard deviation. For this, mean (SD) is used. Using *±* before and after mean is not recommended; summarizing and reporting the data which do not have normal distribution with median, interquartile range, range or both, and reporting confidence interval, their maximum and minimum; reporting variability and distribution of the data use standard deviation, not standard error; illustrating study’s results through tables and graphs instead of lengthy explanations. Tables and graphs should contain informative titles and legends to indicate information; reporting how to calculate amounts of effect and association and their confidence interval of 95%; calculating hazard, amount, and proportion precisely and reporting them with confidence interval of 95% and precision of measurements; reporting and mentioning statistical tests used and expressing one tailed or two tailed of the hypotheses; reporting significance value rounded to two or three decimal places and avoiding abbreviation of *NS* to denote *Not Significant*; reporting first type error value and study’s power; mentioning how to address presuppositions of statistical analysis and observing them in analysis method, for example how to use non parametric tests and regression model; reporting how to control confounding variables and introducing potentially confounding variables in materials and methods; mentioning statistical package or software used for data analysis. The best software for medical research is STATA; mentioning how to deal with missed data and sensitivity analysis, open methods of sampling, post-hoc analysis methods, analysis of subgroups, and exploratory analysis as the most important supplementary and complementary analysis methods in materials and methods; mentioning type of rate (Incidence rate, survival rate), proportion (likelihood, hazard, odds ratio, risk ratio), or risk (absolute, relative, difference in risk), and their confidence interval of 95% precisely; mentioning time period and population unit used to calculate rates; paying attention to important clinical values in difference control for confounding variables; interpreting p value accompanied with important clinical value and paying attention to calculation of number of treatments required to prevent a case of death or complication if needed (NNT); mentioning the name of statistical test used to investigate association and calculating correlation coefficients among variables and reporting the confidence interval of 95%; avoiding *low*, *moderate*, and *high* to report power of associations unless classification of associations are defined and reported; illustrating correlation results through transaction graph. In reporting the amounts of correlation coefficients, paying attention to sample size, value of correlation coefficient and its confidence interval, signal and direction of association and its value of significance; introducing dependent and independent variables in regression analyses, reporting establishment of presuppositions, considering the role of interaction among variables, and offering appropriate model with an acceptable fitness; reporting the method of entering variables into regression model, regression coefficients for each independent variables and their value of significance, model’s goodness-of-fit and the established variance with R-squared statistic and how to validate the model is also important and necessary; converting a non-normal dependent variable into normal through standard error/ robust method instead of logarithm and implementation of conversion on variables; reporting F-test value and its freedom degree in applying ANOVA, and precise value of significance and establishment of presuppositions to employ it; mentioning how to assess and establish model’s presuppositions, introducing time variables till the occurrence of event, the censored, and duration of following up samples, reporting median survival time and amount and proportion of hazard and confidence interval for each variable in the reports relevant to survival analysis, and drawing Kaplan-Meier graph for the model and validating it; reporting criteria of inclusion into and exclusion from the study, how to select samples and patients, and definition of disease; introducing diagnostic tests, kits, instruments, and tools employed to measure variables; paying attention to reliability and validity of the instruments used to gather data, measuring variables, and reporting used measures in this regard and their confidence interval (For example Cronbach’s alpha, ICC); mentioning precise and correct title of the study and its design in materials and methods, how to obtain informed consent from the patients under study, how to do randomization of the groups under study, how to do intervention on the groups in detail and the type of placebo used, how to assess and measure outcomes and how to do blinding and to remove it, how to monitor and evaluate patients and implemented intervention to deal with potential complications and study discontinuation were also considered as technical, important issues which need to be mentioned in materials and methods in a manner appropriate with the type of study.

## Discussion

In this article, the most important points that could improve the quality of scientific, research articles’ methodology in medicine particularly nephrology were gathered and reported. Currently, the manuscripts submitted with the journals are assessed generally in three steps. In the first step, they are assessed by editor-in-chief. In the second step, they are sent to three or four reviewers having authority (preferably working in other universities). After the reviewers’ comments were collected and sent to author(s), if revision in writing is needed, it will be accomplished and the revised manuscript is delivered to editorial board. In this step if the manuscript is approved for publishing, some minor corrections are probably made and the work will be published. These steps which frequently last for some months, could lead to authors’ dissatisfaction. But, the purpose is to increase the quality of published research work. Journals publish some points mainly in first page to show the authors how to adjust the manuscripts or devote special articles to this task. However, serious flaws are still being noted in the methodology of some articles published in journals. Therefore, it is essential to detect defects and offer guideline to promote and enhance articles’ methodology. These points have been mentioned in detail and scattered in the literature (2-32). Each research, scientific journal may offer a summary of these points as an annex. By the findings of our study, authors can, appropriate with their work type and per statistical advice, consult CONSORT checklist in reporting the results of clinical trials, STROBE checklist in observational, epidemiologic studies, standard PRISMA checklist in review articles and meta-analyses, STARD checklist in diagnostic tests and their validation, and COREQ checklist, MECIR checklist of Cochrane institute, and EQUATOR in qualitative research. These checklists are accessible at http://www.equator-network.org. The authors of the present article have used these guidelines in their own studies (34-37). This study recommends that scientific, research journals and medical sciences research centers pay special attention to ten-fold checklists of equator network while holding workshops for empowering researchers in writing articles.

## Conclusion

This study gathered and reported the most important points and factors needing special attention in methodology of medical articles particularly nephrology. Use of these factors by authors, particularly reviewers could lead to improving articles’ and journals’ quality. In addition, use of the points and guidelines of the present study in articles’ materials and methods could avoid research errors.

## Acknowledgments

Hereby, we gratefully thank the members of Department of Epidemiology at Faculty of Health, Shaheed Beheshti University of Medical Sciences for their advice and Mahdi Noroozi, Hossein Lashkardoost, Leila Lashkari, Leila Msasoumzade, and Hamed Ahmadi for offering valuable comments and assistance.

## Authors’ contributions

AA and HS conducted the research. AA prepared the primary draft. HS edited the manuscript.

## Conflict of interests

The authors declared no competing interests.
